# The Determination of Urea

**Published:** 1853-11

**Authors:** 


*The Determination of Urea.* By Professor LIEBIG.—The new method proposed by Liebig has the advantage of simultaneously determining the amount of chlorine and of urea. It is as follows: 100 grammes of mercury carefully purified from bismuth and lead, are dissolved in pure nitric acid; the salt is evaporated to a syrup like consistence, and then enough water is added to reach accurately to 1400 cubic centimetres. Every 100 cubic centimetres contains 7.140 grammes of mercury. If this solution be added to a solution of pure urea, a *snow-white* precipitate falls, which is a compound of urea and oxide of mercury (U+4HgO.) When the urea is all precipitated, and when consequently, nitrate of mercury is the solution, a *yellow* precipitate, hydrated oxide is thrown down by the addition of a little carbonate of soda to a drop of the urea. In order to determine the exact moment when the yellow precipitate occurs, the solution of the nitrate of mercury is of course added drop by drop from a burette, and from time to time a drop of the urine is taken and tested with the soda. One cubic centimetre of the solution corresponds to 10 milligrammes of urea, and from the quantity used, the amount of urea is calculated.

Although nitrate of mercury will thus precipitate urea, the bichloride will not do so, and on this fact is founded the determination of the chlorine. If the solution of urea be not pure, but mixed with chloride of sodium, no precipitate at first occurs with the nitrate of mercury, because sublimate is formed; if the nitrate continue to be added, then at last, the chloride being exhausted, the mercury combines with urea, as in the pure solution. Therefore the quantity of solution used before the white precipitate appears, shows the amount of chlorine which must have combined with the mercury first of all. A cubic centimetre of the mercurial solution corresponds to 10 milligrammes of chloride of sodium.

In testing urine the phosphoric and sulphuric acids are first precipitated with barytic water; and after filtration, the urine is weakly acidified with nitric acid.

Although both the chlorine and the urea *may* be determined by the *same* quantity of urine, it is advisable to use two portions, one for the chlorine and one for the urea. The quantity of nitrate used for the chlorine alone in the first specimen, can be deducted from that used for the urea and chlorine together in the second, and the remainder gives, of course, the quantity which has been used for the urea.—*Brit. and For. Med.-Chir. Review,* from *Annalen d. Chem. und Pharm.,* Band lxxxv. 1853.

